# Furthermore Concerning Propaganda

**Published:** 1920-10-09

**Authors:** 


### FURTHERMORE CONCERNING PROPAGANDA.

Amongst the entertainment advertisements of a morning paper last week I noticed an announcement of a forthcoming dog show. Exceptionally valuable prizes are offered and a large number of entries anticipated. The function is in aid of certain hospitals crippled by debt. Now, I am quite sure that the promoters of this excellent enterprise consider that they are engaged in hospital propaganda, but it would be much more accurate to say that they are encouraging the breeding of good dogs. The hospitals, it is to be hoped, will benefit financially, but the general cause will not be thereby in any respect advanced. It is, indeed, a moot question whether appeals of this kind, constantly repeated, have not a damaging tendency. It happens that the same set of people are victimised every time, and it cannot be wondered if they groan.

### Real and Counterfeit.

Nothing is simpler than to differentiate between real propaganda and that which passes for real. *The educational principle cannot be dissociated from true propaganda.* This is the acid test. All missionary effort comes within this category. Three centuries ago the Roman Catholic Church realised the supreme importance of organised propaganda. In his famous bull, "Inscrutabile," Pope Gregory XV. called into being the Sacred Congregation de Propaganda Fide, which since then has governed the Catholic Missions of the world. The establishment of training-colleges for preparing laborious and pious missionaries is the fundamental function of the Sacred Congregation.

Hospital propaganda in the true sense is, I regret to observe, conspicuous by its absence. The voluntary hospital is a public, not to say a national, institution. Nevertheless it has lived a life of detachment and aloofness.

This statement may probably be contradicted. But I ask the reader to be patient. Take a walk down Regent Street on a fine morning or afternoon when you have a little leisure and need not hurry. Behind those windows crystal clear are Britain's cleverest salesmen, and the admiring pedestrian is like a mouse walking through a maze of traps. New desires within his breast are continually being created simply by beholding the allurements.

Have you ever known the public to be allured into a hospital? Have you known them to be invited there save within very silent limitations? The stock reply will be that a sick room is not a place of entertainment, and that doctors and nurses have something else to do than to take visitors round. I hope to return to this presently, but meantime I want to demonstrate that the attitude of the Regent Street merchant towards the public is very different from that of the hospital *entrepreneur*. The merchant contrives to make you feel that you are conferring on him the favour of his life by deigning to step inside his door. In the hospital, unless you have urgent business, you are *de trop*, and you immediately realise the fact. The consequence is that the wayfarer offers his hospital contribution at the turnstile of the local dog show, or by purchasing inside for half a guinea a plate of cold meat and a crust. He comforts himself that he came as a pigeon to be plucked.

Is it not time that this kind of propaganda, falsely so called, should be superseded? I suggest, at all events, that the time has come to try a new adventure. The fact has to be faced that a very large number of those whose voluntary contributions were in the past the mainstay of the hospital have been forced by altered circumstances to withhold their subscriptions altogether or in part, and that charity entertainments have been overdone. This, however, is not a cause for despair. There are whole millions who have never subscribed, and who have never been asked. So far they have not given the voluntary hospital more than a passing thought.

### A Woman's Question.

To obtain the support of this multitude, regularly and steadily, is the business of the propagandist. Schemes will have to be devised by people of ability and imagination. The traps of Regent Street are laid by trained specialists, and the man in the street is captured, but unaware. He himself believes that he sought out and cleverly discovered a long-felt want. The public must be similarly allured to support the hospitals. There is abundant scope for visitors' entertainment, if I may use the word without being misunderstood. This is an extremely delicate and difficult matter, but the difficulties can be surmounted. In every scheme of propaganda the training of the child is of supreme importance. Tens of thousands of children have never been inside a hospital, whereas every one of them should be sharing the responsibility of maintaining a bed. Giver and receiver would benefit alike. Oh for a Pied Piper!

There is, indeed, no limit to the possibilities. Why not a Hospital Our Day, for example? But first there must be established a propaganda organisation. Is there to be found a Pope Gregory who will call such into being? This presupposes co-ordination and co-operation—a consolidated general interest over and above and distinct from the local and individual interest. IERNE.

